# The survival rate of neonates in Pakistan: Problems in health care access, quality and recommendations

**DOI:** 10.34172/hpp.2022.46

**Published:** 2022-12-31

**Authors:** Muhammad Muzzamil, Maryam Nisa, Shaeroz Raza

**Affiliations:** ^1^Health Services Academy, Islamabad, Pakistan; ^2^Jinnah Medical and Dental College, Karachi, Pakistan

**Keywords:** Neonate mortality rate, Antenatal care, Preterm birth, Infections, Birth defects

## Abstract

There is a high prevalence of infant mortality in South Asia and other parts of Asia, but overall, the bulk of neonatal deaths occur in developing countries. Although Pakistan has made great strides in the past decade to reduce child mortality with the help of foreign donors and the government, very little progress has been made in reducing neonate and infant mortality. Several studies have demonstrated the potential for low-cost therapies to greatly reduce neonatal mortality by helping pregnant mothers and their newborns. We need to shed light on the efforts and problems surrounding this topic in order to find and implement solutions backed by research to lower newborn mortality. This brief overview was produced using international standards for conducting reviews. Researchers opted for an explanatory methodology. Our findings were based on research conducted through PubMed, Google’s literature database, Journals Online, and the Internet Library. All of the works consulted primary sources, such as the World Health Organization (WHO) and the World Bank. The desired findings were obtained by using the term "neonatal mortality." The study’s authors were interested in tracking variations in neonatal mortality over time. The increasing prevalence of neonatal death in Pakistan emphasizes the need for policies and programs that prioritized the health of children. Neonatal survival can be improved with the help of basic obstetric and newborn care in Pakistan.

## Introduction

 Around 65% of all neonatal deaths occur in just 10 countries. Most of these countries are located in Asia. Among these top ten nations, Pakistan ranks third. There are an estimated 300 000 infant deaths annually in the country.^1,2^ According to the most up-to-date data available, the neonatal mortality rate is 42 per 1000 live births,^3^ which accounts for almost 7% of all newborn deaths globally. In comparison to surrounding nations, India has 20 and Afghanistan has 35 per 1000 live births neonatal mortality rate in 2020, according to World Bank data.^4^ Pakistan needs to make substantial improvements in the future years in order to lower the incidence of neonatal mortality when compared to neighboring countries.

 People living in Pakistan often go without necessary medical care because they cannot afford to use the services they need. Multiple factors contribute to this phenomenon, and they become more obvious as one moves from the highest to the lowest wealth quintiles and from one region to another, with the divide between rural and urban areas standing out in particular. Numerous initiatives have been launched over time to improve national maternal and infant health data. However, advancement has been slow, and disparities in things like wealth and income, social standing, literacy levels, and geographical distribution have stayed about the same.^5^

 Under Sustainable Developmental Goal 3 (SDG 3), “Good Health and Well-Being,” the global target for the neonatal mortality rate has been set at 12 per 1000 live births, demonstrating a commitment to ensuring the health of newborns.^6^ In order for Pakistan to improve its neonatal death rate, the country needs extensive reforms on both the policy and execution fronts. According to the reviewed literature, Pakistan has an urgent need for a number of reforms, including initiatives that improve the quality and accessibility of antenatal care (ANC) services for pregnant women and newborns. As there is significant neglect in this area, and as a nation, in the development phase these reforms are necessary to improve the situation.

## Challenges

 The number of neonatal deaths in Pakistan is relatively high. Although the Government of Pakistan has worked to enhance maternal and infant care by strengthening district health systems, encouraging facility-based delivery, and expanding access to prenatal care, the death rate has not been significantly lowered as a result of these attempts. As the government of Pakistan seeks to attain “Universal Health Coverage” by 2030, “the Sustainable Development Goals” present a new opportunity to accomplish the global target of “12/1000” live birth neonate deaths.

 It has been observed that enhancing access alone is not likely to have a significant influence on neonatal outcomes unless it is done in combination with attempts to increase the quality of services. Access to and the quality of care for infants must be enhanced in order to meet the “Every Newborn Action Plan (ENAP)” target of 10 neonate deaths per thousand live births by 2035.^6^

 Several studies have found that a woman’s health has a direct impact on her child’s health. These deaths can be avoided if women’s health issues, particularly those impacting rural women, are addressed by taking into account women’s access to high-quality services and healthcare, as well as by providing skilled birth attendance. Between rural and urban areas, a wide health gap exists. Living in poverty forces women in rural areas to put in more hours of labor than males do because of the lack of economic opportunity. Women’s access to healthcare is hampered by discrimination against women and a lack of access to the broader society as a result of male power dominance. Furthermore, having access to high-quality healthcare appears to be more of a luxury than a basic right in a country where people face food insecurity, must travel great distances to get water, and light their stoves with cow dung. Poor delivery systems, outdated hospitals, and a lack of funds for health care, especially for infant nutrition, all contribute to a lower rate of newborn survival around the world. Despite advancements, there remain inequalities in prenatal care by socioeconomic status and between rural and urban areas. Young, college-educated women, those from financially secure homes, and city dwellers are disproportionately affected.^7^

 However, according to the 2014 Multiple Indicator Cluster Survey, in one Pakistani province, 80% of women had only one trained birth attendant complete a prenatal evaluation. This rate falls to 41% when there is more than one checkup. Even though prenatal care should continue for the entirety of a woman’s pregnancy, many rural Pakistani women still lack access to it: only 19% of pregnant women in these areas receive ANC. The main indicator of maternal and infant health is the percentage of deliveries witnessed by medical professionals. Women in remote and rural Pakistan have fewer resources to pay for competent birth accompaniment, despite the fact that it greatly increases the chances of survival for both the mother and the neonate. In communities that are conservative, impoverished, and conflict-ridden, it is more challenging to secure expert birth attendance. In addition, women’s health suffers from a lack of access to specialist care.^8^

 Infant mortality can be reduced by improving both the availability and the quality of standard treatments. Several developing and middle-income countries have developed their own national certification standards and accreditation systems to regulate and improve healthcare quality. Lacking a national healthcare certification system and comprehensive standards, rules, and practices on quality treatment are two of the most significant challenges to improving health outcomes in Pakistan. Few private and public healthcare providers in Pakistan have voluntarily chosen to implement the ISO 9001: 2008 Quality Management Framework due to the country’s absence of a government template for medical accreditation. Karachi’s Aga Khan University Hospital is the only Pakistani tertiary care academic institution with “Joint Commission International” (JCIA) accreditation. Among the few extra facilities, the “Shaukat Khanum Memorial Cancer Institute and Research Center in Lahore,” the “Shifa International Hospitals in Islamabad,” and the “Rehman Medical Centre in Peshawar” are among the best in quality and patient safety. Just a few examples of the public sector’s exceptional achievements include “the National Program for Family Planning,” “the Sindh Institute of Urology and Transplantation (SIUT),” and “the Peoples’ Primary Healthcare Initiative (PPHI).^9^

## Recommendations

 “End preventable newborn deaths and stillbirths by 2030,” is the title of a report released by UNICEF in July 2020. There are effective, reasonably priced ways to reduce the over 26 million neonatal deaths expected to occur between 2019 and 2030. There has been enormous progress in recent years to improve the health of neonates and reduce death and stillbirths worldwide, even in the countries with the highest rates of neonatal mortality. ENAP recommendation is currently being implemented in 93 countries, with their progress monitored.

 Taking into account these suggestions proves that quick progress is doable. To guarantee that all mothers and their infants receive consistent, high-quality medical care, the ENAP suggests a number of concrete steps that can be taken. To facilitate the rapid collection of this data at the local level, the ENAP is assisting countries in improving their regular health monitoring systems. The ENAP is currently used to record data on premature and unwell newborns, but in the future, standard measurement practices may be used to record these infants’ progress toward their goals. Both programs aim to reduce infant mortality rates, but the Global Action plan also prioritizes reducing the risk of stillbirths.^10^

 According to “The National Health Plan of Pakistan,” inequalities in the delivery of medical services, impediments to healthcare access for the poor, and a focus on inequities relating to equitable maternity, neonatal, and infant healthcare were all underlined (2016-2025). The mission statement asserts that a commitment to fairness must underpin all healthcare operations. As part of an equity-focused plan, it is recommended that particular focus be given to underserved communities in rural areas, inner cities, and other disadvantaged groups. The federal MONHSR&C (Ministry of National Health Services Regulations and Coordination) and the provincial health departments need to work together more closely on MNCH (National Maternal and Newborn Child Health), especially in the areas of monitoring and supervising the care provided to newborns and young infants (NYIs). Funding for the treatment of NYIs should be increased by including it in provincial health departments’ yearly development plans and regular spending.^11^

 Currently, only 0.6% of gross domestic product (GDP) goes toward health care in Pakistan. In order to improve health, education, and other social determinants, we need to significantly expand our investments in these areas. The cost of living has increased faster than funding for primary care services, hence these initiatives have been cut back. Although primary care is supported through the Lady Healthcare Worker program, there has been little investment in strengthening district-level health services, notably the rural health centers and basic health units, despite the fact that these facilities provide the majority of patient care. Health spending at the primary health care level (reproductive, maternal, newborn, child and adolescent health (RMNCAH) & Nutrition) should gradually increase over the next five years, with a focus on equity and contextual needs, and this should be done using a realistic and rigorous method. The federal and provincial governments should work together to create this plan. We also need to distribute funds fairly and stimulate Planning Commission Form-1 (PC1) processing in the area of RMNCH ((Reproductive, Maternal, Newborn, Child and Adolescent Health)-related activities.^12^

 As part of their sustainable development goals, governments should incorporate measures of newborn health and mortality. In low and middle-income countries, it is especially important to provide a secure environment for newborns. The authors argue for a policy in Pakistan’s neonatal health system that places a premium on comprehensive, high-quality aftercare on a national scale. Effective initiatives must be expanded as part of neonatal health policy as saving the lives of infants and reducing neonatal mortality is the focus of the “ENAP”.

## Conclusion

 In Pakistan, where neonatal mortality is growing, health efforts targeting newborns are essential. Pakistan has initiated numerous measures to prevent infant mortality caused by substandard maternal and neonatal health care and a lack of medical professionals. These actions won’t meet the SDGs 2030 minimum aim. Basic obstetric and neonatal care, effective referral, and high-quality follow-up treatment can increase baby survival in Pakistan.

## Author Contributions

**Conceptualization: **Muhammad Muzzamil.

**Writing – Original draft: **Muhammad Muzzamil, Maryam Nisa, Shaeroz Raza.

**Writing – review & editing: **Maryam Nisa, Shaeroz Raza.

## Funding

 None.

## Ethical Approval

 Since only publicly available information was used in this study, no permission from the ethical committee was required.

## Competing Interests

 The authors declare that they have no competing interests.
